# MMP-2 and MMP-9 Polymorphisms and Preeclampsia Risk in Tunisian Arabs: A Case-Control Study

**DOI:** 10.3390/jcm10122647

**Published:** 2021-06-16

**Authors:** Marwa Ben Ali Gannoun, Nozha Raguema, Hedia Zitouni, Meriem Mehdi, Ondrej Seda, Touhami Mahjoub, Julie L. Lavoie

**Affiliations:** 1 Laboratory of Human Genome and Multifactorial Diseases, Faculty of Pharmacy of Monastir, University of Monastir, Monastir 5000, Tunisia; marwabenali24@yahoo.fr (M.B.A.G.); nozharakam@gmail.com (N.R.); hediaztn@gmail.com (H.Z.); touhamimahjoub@gmail.com (T.M.); 2 Laboratory of Histology Embryology and Cytogenetics, Faculty of Medicine, University of Monastir, Monastir 5000, Tunisia; 3 Centre de Recherche du Centre Hospitalier de l’Université de Montréal (CRCHUM), Montréal, QC H2X 0A9, Canada; 4 School of Kinesiology and Physical Activity Sciences, Université de Montréal, Montréal, QC H3T 1J4, Canada; 5 Laboratory of Cytogenetics and Reproductive Biology, Center of Maternity and Neonatology Monastir, Fattouma Bourguiba University Teaching Hospital, Monastir 5000, Tunisia; hbsmahdi@yahoo.fr; 6 The First Faculty of Medicine and General University Hospital, Institute of Biology and Medical Genetics, Charles University, 12800 Prague, Czech Republic; oseda@lf1.cuni.cz

**Keywords:** genotyping, preeclampsia, MMP-9, MMP-2, SNPs

## Abstract

The abnormal production of matrix metalloproteinases (MMPs), especially MMP-9 and MMP-2, plays a pivotal role in hypertensive disorders of pregnancy, and as such, can influence the development of preeclampsia. These alterations may result from functional genetic polymorphisms in the promoter region of MMP-9 and MMP-2 genes, which modify MMP-9 and MMP-2 expression. We investigated the association of MMP-9 polymorphism rs3918242 (-1562 C>T) and MMP-2 polymorphism rs2285053 (-735 C>T) with the risk of preeclampsia. This case–control study was conducted on 345 women with preeclampsia and 281 age-matched women with normal pregnancies from Tunisian hospitals. Genomic DNA was extracted from whole blood collected at delivery. Genotypes for -1562 C>T and -735 C>T polymorphisms were performed using polymerase chain reaction-restriction fragment length polymorphism (PCR-RFLP). An increased frequency of heterozygous MMP-9 -1562 C/T genotype carriers was observed in women with preeclampsia compared to healthy controls (*p* = 0.03). In contrast, the MMP-2 -735 C>T polymorphism was not significantly different regarding frequency distribution of the allele and genotype between healthy pregnant women and women with preeclampsia. Our study suggests that the MMP-9 -1562 C/T variant, associated with high MMP-9 production, could be a genetic risk factor for preeclampsia in Tunisian women.

## 1. Introduction

Preeclampsia (PE) is a multi-system pregnancy-specific disorder classically characterized by elevated maternal blood pressure and proteinuria after 20 weeks of pregnancy. This syndrome complicates 5–7% of pregnancies worldwide and is responsible for 60,000 maternal deaths annually, and a far greater number of fetal and neonatal deaths [1]. Indeed, it is one of the leading causes of maternal and fetal mortality and morbidity [2,3].

PE has been proposed to result from multiple factors, such as angiogenic, inflammatory, and immune response, potentially due to genetic and external environmental factors [4]. However, the role of genetic predisposition is still not well understood, although there is clear evidence of the contribution of family history supported by several segregation and linkage analysis studies, as well as genome-wide association studies [5]. One of the most proposed mechanisms for PE relates to inadequate maternal blood flow to the placenta, caused by impaired spiral artery remodeling, vascular endothelial injury, altered trophoblast cell activity and an exacerbated inflammatory response [6].

Of interest, MMPs are zinc-dependent endopeptidases that degrade different extracellular matrix components and play a role in the remodeling of various tissues [7]. Consequently, MMP activity dysregulation has been reported in clinical conditions affecting the cardiovascular system [8,9], including gestational hypertensive disorders, such as PE [10].

More specifically, MMPs, such as MMP-2 (gelatinase A) and MMP-9 (gelatinase B), have been implicated in endometrial tissue remodeling during estrous cycles and pregnancy [11]. Indeed, MMP-9 and MMP-2 efficiently degrade type IV collagen, a main component of the basement membrane, and are associated with active neovascularization [12,13,14,15]. Of interest, elevated levels of MMP-9 and lower levels of MMP-2 are observed in the serum of women with PE, and similar observations are reported in the umbilical cord plasma of newborns born from women with PE [9,16,17].

MMP-2 and MMP-9 genes contain SNPs, some of which are in the promoter region, that play an essential role in disease development [18,19]. Notably, the MMP-9 -1562 C>T polymorphic substitution, present in this gene’s promoter, is associated with higher transcriptional activity of the gene and higher protein levels of MMP-9 [20]. Interestingly, this T allele has been associated with higher MMP-9 plasma levels in men and women with cardiovascular diseases, such as atherosclerosis and restenosis [21,22], as well as with pregnancy complications [23,24]. More specifically, a few groups have reported an association between this polymorphism and PE risk [23,24,25]. Indeed, six studies have found an association between the -1562 T allele and PE in Brazilian, United Kingdom, Dutch, Polish, and Iranian populations, although in small cohorts (less than 180 cases and 200 controls) [25,26,27,28,29,30]. Hence, it is important to repeat these studies in different populations and large cohorts, as the size of the cohort, ethnic variation and geographical location can contribute to the differences in -1562 T allele distribution.

Concerning MMP-2, its gene is located on chromosome 16. Interestingly, the -735 C>T polymorphism is present in the promoter region and abolishes a Sp1-binding site, leading to decreased promoter activity and reduced MMP-2 expression. The ‘C’ to ‘T’ substitution at the -735 position of the MMP-2 gene may predispose to different conditions, such as increased inflammatory status, tumour metastasis and respiratory diseases [31,32]. However, very few studies have investigated the association between MMP-2 polymorphisms and gestational hypertension and PE [20]. Indeed, only one Iranian study has reported that the maternal -735 T allele is associated with an increased risk of PE in a small cohort (150 cases and 150 controls) [33], while two studies found no association in Brazilian and Polish populations [29,34].

Hence, to date, none of the mentioned MMP polymorphisms have been investigated in the African continent. As genetic variations may differ, depending on ethnicity [35,36], the present study aimed to evaluate the association of MMP-2 -735 C>T (rs2285053) and MMP-9 -1562 C>T (rs3918242) polymorphisms with the risk of PE in a large Tunisian cohort (281 controls and 345 cases).

## 2. Materials and Methods

### 2.1. Subjects

This retrospective case–control study, which we previously described in detail [37,38], involved 345 unrelated Tunisian women with PE, who were recruited between May 2012 and June 2013 from the gynecology service (hospitalized and outpatient) of Farhat Hached University Hospital (Sousse, Central Tunisia), Fattouma Bourguiba University Hospital (Monastir, Central Tunisia), Taher Sfar University Hospital (Mahdia, Eastern Tunisia), and Gafsa Hospital (Southern Tunisia). The inclusion criteria were PE during a natural pregnancy, defined as gravid hypertension, assessed as systolic blood pressure (SBP) > 140 mmHg, diastolic blood pressure (DBP) > 90 mmHg, a rise in SBP > 30 mmHg, or DBP > 15 mmHg on at least two measurements, 6 h apart, and significant proteinuria ≥ 300 mg/24 h after 20 weeks of gestation [2]. Severe PE was defined as SBP ≥ 160 mmHg or DBP ≥ 110 mmHg and proteinuria ≥ 500 mg/24 h [2]. As subject recruitment was conducted in 2012, we used the definition and classification of PE determined by the American College of Obstetricians and Gynecologists (ACOG), which was active at the time [2]. This is in contrast with the actual ACOG guidelines, where PE is diagnosed by the presence of de novo hypertension after 20 weeks of gestation, accompanied by proteinuria and/or evidence of maternal acute kidney injury (AKI), liver dysfunction, neurological features, hemolysis or thrombocytopenia, or intra-uterine growth restriction [39]. Women who met the criteria for PE, but not severe PE, were defined as moderate PE. Our study included women with previously diagnosed chronic hypertension and a history of PE.

We recruited women with normal pregnancies from the same geographical area and without any obstetrical complications for the control group. Exclusion criteria for this group were known personal or family history of hypertension and PE. The pairing of women with normal pregnancy was done on the basis of the age of preeclampsia cases (+/− 1 year of age). Hence, certain women with normal pregnancies were not chosen based on their age. Local ethics committees approved the study protocol, and both PE cases and control women gave written informed consent for participation in the study. The study participants’ demographic and clinical data were collected from a designed questionnaire and medical records, as done previously [40].

### 2.2. MMPs Genotyping

Genotyping of MMP-2 -735 C>T and MMP-9 -1562 C>T SNPs was performed on DNA extracted from blood samples by the proteinase K/salting-out method [41], using PCR-restriction fragment-length polymorphism (PCR-RFLP) method [42]. The target fragments containing these two polymorphisms were amplified using the following primers: for the -735 C>T: 5′-GGATTCTTGGCTTGGCGCAGGA-3′ (forward) and 5′-GGGGGCTGGGTAAAATGAGGCTG-3′ (reverse); for the -1562 C>T polymorphism: (forward) 5′-GCCTGGCACATAGTAGGCCC-3′ (forward) and 5′-CTTCCTAGCCAGCCGGCATC-3′ (reverse). For PCR amplification, the reaction mixture consisted of 2.5 μL of DNA sample, 0.5 µM of each primer, 10× PCR buffer, 1.5 mM of MgCl2, 0.5 U of Taq DNA polymerase (Invitrogen), 0.2 mM of each dNTP and water was added to obtain a final volume of 20 μL. To detect polymorphisms, the samples were denatured at 95 °C for 5 min, followed by 35 cycles of denaturation at 94 °C for 30 s, annealing at 59 °C for 30 s and extension at 72 °C for 30 s. The samples were incubated at 72 °C for an additional 5 min for the final extension. Amplicons were 436 and 391 bp for -1562 C>T and -735 C>T, respectively. The SphI and HinfI restriction enzymes produced 242 and 194 bp fragments for the -1562 T allele and 338 and 53 bp fragments for the -735 T allele. The fragments were analyzed on a 2% agarose gel.

### 2.3. Statistical Analysis

Continuous data are expressed as mean ± standard deviation (SD) and were compared using the Mann–Whitney U-test. Categorical variables are presented as numbers (percentages of total) and were compared using the Chi-square test (χ^2^). Allele frequencies were calculated by the gene-counting method, and each polymorphism was tested for Hardy–Weinberg equilibrium using χ^2^ goodness-of-fit test using HPlus 2.5 software. The putative predictors of PE, the clinical factors of study participants, and the two polymorphisms studied were initially evaluated by univariate analysis and then by multivariate logistic regression analysis. We calculated the corresponding crude odds ratio (cOR) and its 95% confidence interval (95% CI) and then the adjusted odds ratio (aOR) and its 95% CI; the main covariates that we adjusted for were gestational age, BMI, delivery method and baby weight. Statistical significance was set at *p* < 0.05.

## 3. Results

### 3.1. Study Subjects

Demographic and clinical features of PE cases and control women are shown in Table 1. According to the defined criteria, 162 of the 345 women had severe PE. Among them, 87 (25.2%) developed a severe early onset form (before 34 weeks of gestation); there were 15 cases of eclampsia, but no cases of HELLP (hemolysis, elevated liver enzymes, low platelets) syndrome. As expected, women with PE had significantly elevated SBP and DBP, higher BMI, and gave birth at a lower gestational age. Not surprisingly, women with PE had a significantly higher incidence of primiparous pregnancies, as this is a known risk factor for this disease. In addition, we found that babies born from women with PE had significantly lower weights. Accordingly, gestational age, BMI, delivery method and baby weight were selected as covariates controlled for in the subsequent analysis.

### 3.2. Association Studies

Minor allele frequencies of MMP-9 -1562 T and MMP-2 -735 T were not significantly different among PE cases and control women (Table 2). Setting the major homozygous genotype C/C as a reference (OR = 1.00), significantly higher frequencies of heterozygous MMP-9 -1562 C/T genotype carriers were observed in PE cases compared to control women (*p* = 0.03; OR (95% CI) = 1.62 (1.03–2.56)) (Table 3). We found no significant differences in the distribution of the remaining genotypes between PE cases and control women.

### 3.3. Association Analysis

Our results showed a lack of association between the tested MMP-9 and MMP-2 polymorphisms and the severity of PE, irrespective of the genetic model used (co-dominant, dominant, or recessive) (Table 4). These associations remained not significant, even after adjustment for gestational age, BMI, delivery method and baby weight.

## 4. Discussion

To our knowledge, in the African continent, this is the first case–control study that investigated the association of polymorphisms in the promoter region of MMP-2 (-735 C>T) and MMP-9 (-1562 C>T) with the risk of PE. More specifically, these polymorphisms were investigated in a large Tunisian Arab cohort (281 controls and 345 cases), compared to most studies, which studied small cohorts. As such, in our study, we may have higher statistical power for the genetic analysis. Notably, the CT genotype of MMP-9 -1562 C>T was associated with an increased risk of PE in our Tunisian cohort. However, the frequency distribution of MMP-2 -735 C>T allele and genotype polymorphism was not associated with PE.

In contrast to our results, most studies have reported no association between the -1562 C/T polymorphism and the risk of PE in the Netherlands [30], Brazilian [25,29], United Kingdom [27], Poland [26] and Iranian [28] populations. However, PE cases with chronic hypertension and a history of PE were excluded in these reports, in contrast to our study. Only one study, conducted by Rahimi et al., found an association with the MMP-9 -1562 C>T polymorphism but only found in women with severe PE. Hence, these differences may result from the cohort size of most of these studies as our cohort is almost twice the size of these other studies. As such, they may have been underpowered to find any MMP-2 and MMP-9 polymorphism association with PE. Importantly, they investigated a heterogeneous population. As such, ethnic variations and geographical location could have contributed to the lack of association.

With regard to the lack of association of the polymorphism MMP-2 -735 C>T with PE, these are in agreement with two previous studies which looked at this SNP in Brazilian [34] and Polish [26] populations. However, another investigation conducted on 144 women with PE and 103 healthy control subjects in Iran found an association between the MMP-2 -735 T polymorphism and PE risk [33]. Interestingly, in this Iranian study, all women had a Kurdish ethnic background and women with chronic hypertension were excluded from their cohort. As such, this may have contributed to a more homogenous cohort and allowed them to uncover this association. As such, the inclusion of women with chronic hypertension in our study and our unique geographical location and ethnicity may have contributed to the lack of association in our study.

Mechanistically, changes in MMP expression and activity may lead to increased oxidative stress and inflammatory mediators, which are associated with endothelial dysfunction and, in turn, contribute to the pathogenesis of PE [43,44]. More specifically, in healthy pregnancies, MMPs, such as MMP-9 and MMP-2, play a crucial role in the process of trophoblast cell invasion, remodelling spiral arteries as well as in angiogenesis [7]. In contrast, in women with PE, high MMP-9 activity has been implicated in the pathological remodelling of the extracellular matrix of the arterial wall by causing an accumulation of collagen [45]. MMP-2 and MMP-9 dysregulation could lead to altered endometrial matrix degradation and impaired trophoblastic invasion and placentation, thus, inducing PE symptoms [15,16]. As the MMP-9 -1562 C/T and MMP-2 -735 C/T polymorphisms are located in the promoter region, they may regulate the protein production of these MMPs [26]. Interestingly, the presence of the T allele of the MMP-9 -1562 C>T polymorphism is associated with higher transcriptional activity of the gene and elevated MMP-9 protein levels in biological fluids and tissues [46]. Additionally, it has been reported that MMP-9 protein levels in the umbilical cord arterial wall and the plasma of newborns from preeclamptic pregnancies are increased compared to those from women with normal pregnancies [10].

In contrast to the study by Rahimi et al., we found a lack of association between the tested MMP-9 and MMP-2 variants and PE severity. Indeed, they found a significantly higher frequency of the MMP-9 CT + TT genotypes among women with severe PE [45]. This discordance may be due to the small number of cases of women with severe PE in our cohort or may relate to differences in PE subtypes and other ethnic/racial factors in the study population. Moreover, this result was irrespective of the genetic model used (co-dominant, dominant, or recessive) and remained insignificant, even after adjustment for clinical parameters (gestational age, BMI, delivery method and baby weight).

Finally, the differences observed between our results and those previously published may stem from ethnic and racial variations in the distribution of MMP variants. Additional studies in large cohorts from other ethnic groups will be required to confirm this speculation. One of the strengths of our study is the homogeneity of the population. This minimized the differences in genetic background, inherent to gene association studies and the possibility of ethnic stratification on the distribution of the MMPs allele and genotype, as well as the potential covariates to control. A limitation of the present case–control study was that we did not correlate genotypic data with MMP plasma levels. Follow-up studies are needed to analyze other variants in these genes and assess if altered MMP activity in PE pathogenesis.

## 5. Conclusions

In conclusion, MMP-9 promoter polymorphism could influence the risk of PE development through increased production of MMP-9 protein in the maternal circulation and at the maternal–fetal interface. Our results indicate, for the first time, that such an association is present in a Tunisian population where we found that the carriage of the heterozygous MMP-9 -1562 C/T genotype, which is associated with higher MMP-9 production, was associated with PE risk.

## Figures and Tables

**Table 1:** Demographic and clinical characteristics of controls and patients.

Characteristic		Controls (*n* = 289)	Cases (*n* = 345)	*p* ^1^
Age (years) ^2^		30.5 ± 5.8	31.3 ± 7.0	0.121
BMI (kg/m^2^) ^2^		28.6 ± 4.2	32.2 ± 5.0	<0.001
Newborn weight (g) ^2^		3253.3 ± 405.2	2888.0 ± 755.7	<0.001
GA at blood sampling ^2^		38.2 ± 3.0	35.8 ± 3.6	<0.001
Region ^3^	Sahel region	199 (66.3)	234 (78.0)	<0.001
	Central Tunisia	7 (2.3)	33 (11.0)	
	Southern Tunisia	94 (31.3)	33 (11.0)	
Blood pressure (mmHg) ^2^	Systolic	112.3 ± 9.3	155.4 ± 14.9	<0.001
	Diastolic	68.8 ± 7.9	95.0 ± 8.7	
Delivery method ^3^	Vaginal delivery	188 (62.7)	165 (55.0)	0.068
	Caesarian sections	112 (37.3)	135 (45.0)	
Pregnancy status ^3^	Multiparous	164 (54.7)	75 (25.0)	<0.001
	Primiparous	131 (43.7)	222 (74.0)	
	Nulliparous	5 (1.7)	3 (1.00)	
Chronic hypertension		0 (0.0)	155 (39.1)	<0.001

^1^ Student *t*-test for continuous variables, and Pearson’s chi-square for categorical variables. ^2^ Mean ± SD. ^3^ Number (percent total). BMI, body mass index; GA, gestational age.

**Table 2:** Distribution of MMP-9 and MMP-2 alleles in PE cases and control women.

Gene (SNP)	Position ^1^	MAF	Patients	Controls	χ^2^	*p* Value	OR (95% CI)
MMP-9 (-1562 C>T)	chr8:81279768	T	64 (18.6) ^2^	38 (13.5)	2.87	0.09	0.72 (0.50–1.05)
MMP-2 (-735 C>T)	chr16:55478465	T	78 (23.5)	53 (18.9)	1.94	0.16	0.80 (0.58–1.09)

^1^ Location on the chromosome. ^2^ Number of alleles (frequency). MMP: Matrix Metalloproteinases; PE: preeclampsia; SNP: single-nucleotide polymorphism; MAF: minor allele frequency; T: threonine; chr: chromosome.

**Table 3:** Genotypic frequencies of the studied polymorphisms.

Gene (SNP)	Genotype	Controls ^a^	Cases ^a^	*p*	*p* ^c^	OR (95% CI)
**MMP-9** **(** **-** **1562 C>T** **)**	C/C	243 (86.7) ^b^	281 (81.4)	**0.03**	0.14	1
	**C/T**	**33** **(11.7)**	**62** **(17.9)**			**1.62** **(1.03–2.56)**
	T/T	5 (1.7)	2 (0.5)			0.35 (0.07–1.80)
MMP-2 (-735 C>T)	C/C	228 (81.1)	254 (76.5)	0.26	0.33	1
	C/T	41 (14.6)	65 (19.6)			1.42 (0.93–2.19)
	T/T	12 (4.3)	13 (3.9)			0.97 (0.43–2.17)

^a^ Study subjects included 345 PE cases and 281 control women. ^b^ Number of genotypes/subjects (percent total). ^c^ *p*-values adjusted for age, gender, diabetes, smoking, hypertension.

**Table 4:** Association of MMP-9 and MMP-2 genotypes with the severity of PE.

Model	Genotype	Genotype Distribution MMP-9				Genotype Distribution MMP-2			
		Moderate PE *n* = 183	Severe PE *n* = 162	*p*	*p* ^b^	Moderate PE *n* = 178	Severe PE *n* = 154	*p*	*p* ^b^
Co-dominant model	C/C	150 (82) ^a^	131 (80.9)	0.22	0.29	132 (74.2)	122 (79.2)	0.39	0.65
	C/T	33 (18)	29 (17.9)			37 (20.8)	28 (18.2)		
	T/T	0 (0)	2 (1.2)			9 (5.1)	4 (2.6)		
Dominant model	C/C	150 (82)	131 (80.9)	0.79	0.17	132 (74.2)	122 (79.2)	0.28	0.39
	C/T-T/T	33 (18)	31 (19.1)			46 (25.8)	32 (20.8)		
Recessive model	T/T	0 (0)	2 (1.2)	0.08	0.6	9 (5.1)	4 (2.6)	0.24	0.97
	C/C-C/T	183 (100)	160 (98.8)			169 (94.9)	150 (97.4)		

^a^ Number of genotypes/subjects (percent total). ^b^ *p*-values adjusted for gestational age, BMI, delivery method and baby weight.
